# Associations between bacterial genotype and outcome of bovine clinical *Staphylococcus aureus* mastitis

**DOI:** 10.1186/1751-0147-56-2

**Published:** 2014-01-08

**Authors:** Åsa Lundberg, Anna Aspán, Ann Nyman, Helle Ericsson Unnerstad, Karin Persson Waller

**Affiliations:** 1 Department of Animal Health and Antimicrobial Strategies, National Veterinary Institute, Uppsala SE-751 89, Sweden; 2 Department of Clinical Sciences, Swedish University of Agricultural Sciences, Box 7054, Uppsala SE-750 07, Sweden; 3 Department of Bacteriology, National Veterinary Institute, Uppsala SE-751 89, Sweden

**Keywords:** *Staphylococcus aureus*, Dairy cow, Clinical mastitis, Genotypes, Somatic cell count, Long-term mastitis outcome, Pulsed-Field Gel Electrophoresis, PFGE

## Abstract

**Background:**

*Staphylococcus aureus* is an important cause of clinical mastitis in dairy cows worldwide. The cure rate after antimicrobial treatment of clinical *S. aureus* mastitis is very variable due to both cow and bacterial factors. Studies have shown that bacterial genotype might affect short-term bacteriological and clinical cure, but the long-term outcome has been less studied. The objectives of this study were to investigate associations between bacterial genotype and long-term outcome of veterinary-treated clinical mastitis (VTCM) caused by *S. aureus* during a follow-up period of 120 days and to study genotype variation among Swedish *S. aureus* isolates. *S. aureus* isolates from cases of VTCM were genotyped by pulsed-field gel electrophoresis. Long-term outcome measurements used were somatic cell count (SCC), additional diagnoses of VTCM, milk yield and culling. Isolates were classified into clusters (>80% similarity) and pulsotypes (100% similarity). Clusters and pulsotypes were grouped according to occurrence. Multivariable mixed-effect linear regression models including cow and bacterial factors with possible influence on SCC or milk yield were used to calculate differences in SCC or milk yield between groups. Additional outcome measures were calculated using a test of proportions.

**Results:**

The isolates (n = 185) were divided into 18 clusters and 29 pulsotypes. Two pulsotypes were classified as common, and were found in 64% of the cases of VTCM. Remaining isolates were classified as less common or rare pulsotypes. The distribution was similar at cluster level. Outcome was calculated from follow-up data on 111 cows. Significantly lower SCC during the follow-up period was found in cows infected with common clusters compared to in cows infected with less common/rare clusters. The proportion of cows with SCC <200 000 cells/ml during the whole follow-up period was significantly higher in the group common clusters than in the group less common/rare clusters. Bacterial genotype did not influence the other outcome parameters.

**Conclusions:**

In Sweden, two *S. aureus* pulsotypes, identified in about 64% of clinical *S. aureus* cases, were widespread. Cows infected with the common genotypes had significantly lower SCC during 120 days after treatment compared to cows infected with less common or rare genotypes.

## Background

*Staphylococcus aureus* is an important udder pathogen causing intramammary infections in dairy cows worldwide. It is a common finding in clinical mastitis in many countries [1-3] and in a national survey performed in Sweden in 2002–2003, *S. aureus* was found to be the most common pathogen, accounting for 21.3% of all cases of clinical mastitis [4]. The present study is based on *S. aureus* isolates identified in that survey.

Many cases of clinical *S. aureus* mastitis are treated with antimicrobials, but the cure rate is very variable due to a variety of cow and bacterial factors such as parity of the cow, chronicity of the infection and β-lactamase production of the pathogen [5-8]. Another feature that could influence the cure rate is bacterial genotype. The introduction of molecular bacteriology has revealed that clinical *S. aureus* mastitis can be caused by a large variety of bacterial genotypes [9-12]. A number of methods for genotyping of *S. aureus* have been described. Two methods often used are multi-locus sequence typing (MLST) and pulsed-field gel electrophoresis (PFGE). MLST has the advantage of generating results that are easily comparable between laboratories. However, PFGE remains a good choice for research purposes with excellent typeability, discriminatory power and easy interpretation [13,14]. In addition, the use of PFGE enabled us to include a sub-set of samples from the survey previously genotyped [15]. In that study, a large number of *S. aureus* pulsotypes were found when comparing a selection of isolates from three Swedish regions [15]. It was clear, however, that some pulsotypes were more common than others. The predominance of certain genotypes has also been observed elsewhere [10,16,17].

Differences between bacterial isolates of different genotypes in clinical signs [11,18-21] and persistence of infection [19,22] have also been observed. Moreover, the likelihood of cure after antimicrobial therapy has also been shown to differ between genotypes of *S. aureus.* Haveri *et al.*[19] found that infection with one genotype was related to severe symptoms, but the same strain was also invariably eliminated from the udder after treatment with antimicrobials. In addition, Dingwell *et al.*[23] found that some genotypes of *S. aureus* may be more likely to be eliminated by dry cow treatment than other genotypes. In addition, van den Borne *et al.*[24] found that host adaptation influenced bacteriological cure after antimicrobial treatment during lactation. The latter two references did, however, only include subclinical cases of mastitis.

In the above mentioned studies on the significance of genotype differences on the outcome of antimicrobial treatment of *S. aureus* mastitis, the focus has been on short-term bacteriological and/or clinical cure [19,23,24]. When evaluating cure of *S. aureus* mastitis it is, however, important to include both short-term and long-term outcome. Therefore, studies on differences between *S. aureus* genotypes in long-term outcome, using for example somatic cell count (SCC), number of new episodes of clinical mastitis, milk production, and culling are warranted. The identification of genotype differences in recovery may result in better tools for selection of cases for antimicrobial treatment. This could be economically favourable and minimize the use of antimicrobials.

The main aim of this study was to investigate associations between bacterial genotype and long-term outcome of veterinary-treated clinical mastitis (VTCM) caused by *S. aureus*. In order to investigate this, isolates from naturally occurring cases of clinical mastitis included in the national survey mentioned above [4] were genotyped using PFGE. Outcome measurements used were cow composite milk SCC, additional registered VTCMs, daily milk yield, and culling rate during a follow-up period of 120 days. Another aim was to gather more comprehensive knowledge about genotype variation among Swedish *S. aureus* isolates.

## Methods

### Isolates

*Staphylococcus aureus* isolates (n = 223) were obtained from cases of VTCM collected in 2002–2003 when a national survey on the distribution of udder pathogens was performed [4]. A case was defined as a cow with macroscopically altered milk in one or more quarters, with no previous episodes of clinical mastitis in the current lactation or antimicrobial treatments during the preceding 30 days and with a composite milk SCC of <200 000 cells/ml at the latest monthly milk recording, when such information was available. Moreover, the herds from where the cases came had to be enrolled in the Swedish Official Milk Recording Scheme (SOMRS; Swedish Dairy Association, Stockholm).

Milk samples were taken aseptically from affected udder quarters by the veterinary practitioner. Ten microlitres of milk were cultured on 5% bovine blood agar plates, and the agar plates were incubated at 37ºC for 16–24 hours. The plates were evaluated at the veterinary practice and then sent to the National Veterinary Institute (SVA), Uppsala, Sweden, for further investigations according to accredited routines [4]. At SVA, *S. aureus* was identified by colony morphology and the presence of haemolysis. Isolates with typical morphology and both complete and incomplete haemolysis were considered *S. aureus*. A tube coagulase test was performed when there was no haemolysis or when only a zone of complete haemolysis was present. Coagulase-positive isolates were considered *S. aureus*. All isolates were tested for β-lactamase production by the clover-leaf method [25], stored in trypticase soy broth containing 15% glycerol and frozen.

### Genotyping by pulsed-field gel electrophoresis

After thawing, isolates were cultured twice overnight at 37ºC on 5% bovine blood agar supplemented with 0.05% esculine. Pulsed-field gel electrophoresis was carried out using the same protocol as Capurro *et al.*[15]. Macrorestriction patterns were analyzed using Bio-Numerics (version 6.6; Applied Maths, Inc.). Seventy-six of the isolates were pulsotyped in a previous study [15] and their macrorestriction patterns were added to the present study. Isolates were divided into clusters where a maximum of three bands differed in the macrorestriction pattern (similarity level of about 80% or more). Clusters were subdivided into pulsotypes with a higher discriminatory power. Isolates were considered to be of the same pulsotype when the number and positions of bands in the macrorestriction pattern were the same (100% similarity level). Clusters (C) were identified by Arabic numerals (C1, C2 etc.) and pulsotypes within each cluster were identified with lower-case letters (C1a, C1b etc.).

### Outcome measurements and cow records

Somatic cell count, recurring or new cases of VTCM, daily milk yield, and culling due to mastitis were used as outcome measurements. Cow composite SCC and daily milk yield from monthly milk recordings during the follow-up period was collected from the SOMRS. Disease recordings were collected from the Swedish Animal Disease Recording System (SADRS) via the SOMRS. An additional diagnosis of VTCM (VTCMadd) in the same cow (quarter level data not available) was defined as an additional record of VTCM in the SADRS from 14 to 120 days after the initial VTCM. Unfortunately, not all cases of clinical mastitis are recorded in the SADRS [26]. Therefore, the absence of a VTCM for a particular cow in the follow-up period could not automatically be counted as a negative for VTCMadd. However, it was supposed that records from SADRS for the individual cow were complete if there was a disease recording in the database for the case of VTCM from which the sample was sent in. For all cows, date and cause of culling was gathered from the SOMRS. The cow was considered to have been culled due to mastitis if the recorded primary or secondary reason for culling was either mastitis or increased somatic cell count. All outcome measurements were considered for the follow-up period of up to 120 days after VTCM. Due to few recordings of culling and VTCMadd, a combined outcome measure called “treatment failure” was created for cows that had a VTCMadd registered and/or that were culled due to mastitis.

In addition, cow data including breed, parity, and days in milk (DIM) was collected from the SOMRS.

### Data editing and statistical analyses

To ensure that the outcome variables were not affected by the presence of more than one pathogen when evaluating outcome of VTCM, cows that had more than one infected udder quarter or multiple pathogens present in the same quarter were excluded from the original 223 isolates. In addition, one cow was excluded due to missing cow identity. From the remaining cows, only the first submitted isolate from each herd was included to ensure epidemiological independence among isolates. This resulted in a total of 135 cows to follow up. Seven cows were dried off during the follow-up period. For these cows, only data from test milkings before dry-off was included.

To study genotypic variation in Sweden, cows were included independent of number of infected udder quarters and presence of additional pathogens. Again, only the first submitted isolate from each herd was included to ensure epidemiological independence among isolates, and one sample was excluded due to missing cow identity. Thus, descriptive statistics on occurrence of different genotypes was based on a total of 187 isolates.

Because of the relatively small study material, genotypes with few contributing isolates had to be grouped to achieve statistical power. We hypothesized that rare genotypes might have other characteristics than the more common ones. Common clusters (more than 20 isolates per cluster) were analyzed both individually and as a group, less common clusters (3–19 isolates per cluster) were analyzed as one group, and rare clusters (1–2 isolates per clusters) formed another group. Pulsotype isolates were categorized into pulsotype groups based on the same criteria.

To test for significant differences in SCC or milk yield (kg/day) between clusters, between cluster groups, between pulsotypes, and between pulsotype groups, univariable mixed-effect linear regression models were constructed. Repeated measurements of SCC or milk yield within cow, for the follow-up period of 120 days after VTCM, were used as outcome, and genotype (clusters or pulsotypes or groups of these) as the predictor variable. Any significant differences between genotypes or genotype groups were then further tested using multivariable mixed-effect linear regression models that were developed to take into account other parameters that can influence the SCC or milk yield. Like the univariable models, these models were also constructed with repeated measurements within cow of the dependent variable SCC or milk yield, for the period up to 120 days after VTCM. The cow parameters breed, parity, milk yield (used in SCC model), SCC (used in milk yield model) and DIM at monthly milk recordings, and the bacterial parameters cluster or pulsotype and β-lactamase production, were included as independent variables. A manual stepwise backward model selection procedure was used to reduce the full models (including all independent variables considered), and variables with a p-value of ≤0.05 were retained in the model. Biologically plausible interactions between the main effects were tested and were retained in the model if the p-value was ≤0.05. To obtain normally distributed residuals, SCC was transformed using the Box-Cox power transformation ((SCC^-0.0816351^-1)/-0.0816351). To confirm the correctness of the transformation, the normality of the residuals was analyzed using a quantile-quantile plot and by plotting residuals against the fixed effect predictions after the analyses.

In addition, differences in proportions of cows with a SCC <200 000 cells/ml at all test milkings from day 14 to 120 between the cluster groups common clusters and less common/rare cluster were tested using a two-sample test of proportions with 95% confidence interval. The cut-off of 14 days was chosen to allow for SCC to return to normal after successful treatment and only cows with measurements from at least two milk recordings were included in the calculations.

The difference in occurrence of β-lactamase production between common clusters and less common/rare clusters was evaluated using the Fisher’s exact test.

Descriptive statistics were used to summarize data on treatment failure. In addition, differences in treatment failure between cluster groups were calculated using a two-sample test of proportions with 95% confidence interval.

All statistical analyses were performed using Stata Software (StataCorp., 2010; Stata Statistical Software: Release 11.2; College Station, TX, USA: StataCorp LP.).

## Results

### Distribution of genotypes

Of the 187 investigated *S. aureus* isolates included in this part of the study, PFGE generated results for 185 isolates, which were divided into 18 clusters (C1-C18) and 29 pulsotypes (Table 1). The most common clusters were C11, accounting for 44% of the isolates, and C15, accounting for 29% of the isolates. Clusters C1, C3, C10 and C17 were considered less common clusters, represented by 5–7 isolates each. The remaining isolates belonged to rare clusters, together accounting for 7% of all isolates. Each cluster could be divided into 1 to 3 pulsotypes. C11a was the most prevalent pulsotype, accounting for 43% of the isolates, followed by C15b. C11a and C15b together accounted for 64% of the isolates. Twelve percent of the isolates belonged to rare pulsotypes, represented by one or two isolates each. The remaining isolates were considered less common pulsotypes and belonged to one of seven pulsotypes, each represented by three to 19 isolates.

**Table 1:** **Cluster and pulsotype prevalence (numbers (n) and proportions (%)) of *Staphylococcus aureus *** **isolates (n = 185) from cases of veterinary-treated clinical mastitis**

**Cluster**	**n**	**%**	**Cluster group**^**1**^	**Pulsotype**	**n**	**%**	**Pulsotype group**^**1**^
C1	6	3.2	Less common	C1a	3	1.6	Less common
				C1b	1	0.5	Rare
				C1c	2	1.0	Rare
C2	1	0.5	Rare	C2a	1	0.5	Rare
C3	18	9.7	Less common	C3a	17	9.2	Less common
				C3b	1	0.5	Rare
C4	1	0.5	Rare	C4a	1	0.5	Rare
C5	1	0.5	Rare	C5a	1	0.5	Rare
C6	1	0.5	Rare	C6a	1	0.5	Rare
C7	1	0.5	Rare	C7a	1	0.5	Rare
C8	2	1.0	Rare	C8a	2	1.0	Rare
C9	1	0.5	Rare	C9a	1	0.5	Rare
C10	5	2.7	Less common	C10a	3	1.6	Less common
				C10b	1	0.5	Rare
				C10c	1	0.5	Rare
C11	82	44.3	Common	C11a	79	42.7	Common
				C11b	1	0.5	Rare
				C11c	2	1.0	Rare
C12	1	0.5	Rare	C12a	1	0.5	Rare
C13	1	0.5	Rare	C13a	1	0.5	Rare
C14	1	0.5	Rare	C14a	1	0.5	Rare
C15	54	29.2	Common	C15a	8	4.3	Less common
				C15b	40	21.6	Common
				C15c	6	3.2	Less common
C16	1	0.5	Rare	C16a	1	0.5	Rare
C17	7	3.8	Less common	C17a	3	1.6	Less common
				C17b	3	1.6	Less common
				C17c	1	0.5	Rare
C18	1	0.5	Rare	C18a	1	0.5	Rare

^1^Common = more than 20 isolates per cluster or pulsotype. Less common = 3–19 isolates per cluster or pulsotype. Rare = 1–2 isolates per cluster or pulsotype.

### β-lactamase production

Of the 185 investigated *S. aureus* isolates included in this part of the study, 6.5% produced β-lactamase. The proportion of such isolates was lower (p < 0.001) among common clusters (1.5%) than among less common/rare clusters (20.5%).

### Descriptive statistics of the material

Complete data about breed, parity and lactation stage was available for 169 of the 185 cows with isolates included in the genotyping. Forty-eight percent of the cows were Swedish Holstein, 46% were Swedish Red, and 6% were of other breeds or cross-breads. Half of the cases of VTCM occurred during the first month of lactation, while the remainder was spread out during the rest of the lactation. Thirty-five percent of the isolates came from first-parity cows, 20% from second-parity cows, 21% from third-parity cows and 24% from fourth parity cows or older.

### Outcome as measured by SCC and milk yield

Of the 135 *S. aureus* isolates available for the outcomes, PFGE generated results for 133 isolates from 133 cows. Of these, monthly milk recordings after the VTCM were registered for 111 cows. In total, 374 monthly SCC and milk yield recordings, in average 3.4 recordings per cow, were registered for these cows. Missing data was due to the cow being culled before first test-milking, the cow being close to dry off at VTCM, or records missing altogether. One SCC value, at 119 days after VTCM from a cow infected with C15b, was considered an extreme outlier as the SCC was very high (>9 999 000/ml). Since such high SCC is not expected in a cow without clinical symptoms, and no VTCMadd was recorded, it was supposed that the disease recordings for the cow were incomplete or that there was a problem with the SCC analysis or the data handling. This extreme outlier SCC was therefore excluded.

**Figure 1:**
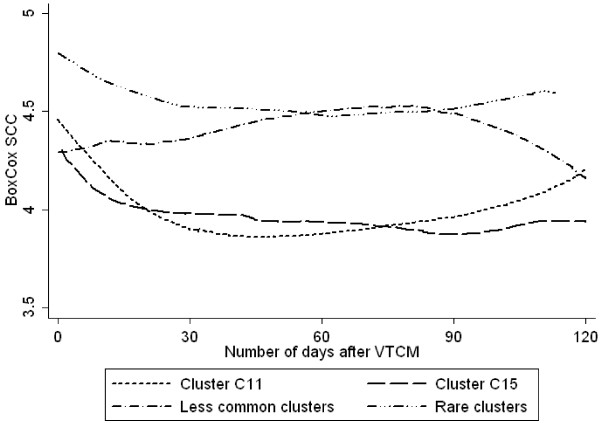
**Long-term outcome as measured by SCC.** Cow somatic cell count (BoxCox transformed SCC^-0.0816351^-1)/-0.0816351; n = 111 cows) at test milkings up to 120 days after veterinary-treated cases of clinical mastitis caused by *Staphylococcus aureus* of different clusters/cluster groups.

**Figure 2:**
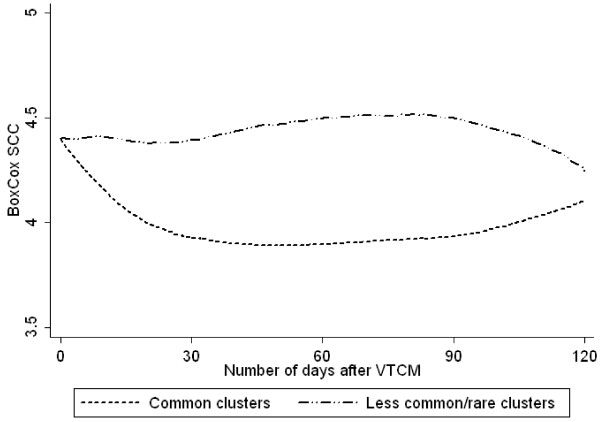
**Long-term outcome as measured by SCC; common clusters combined and less common/rare clusters combined.** Cow somatic cell count (BoxCox transformed SCC^-0.0816351^-1)/-0.0816351; n = 111 cows) at test milkings up to 120 days after veterinary-treated cases of clinical mastitis caused by *Staphylococcus aureus* of common clusters compared with those caused by less common/rare *Staphylococcus aureus* clusters.

The 111 isolates originating from above mentioned 111 cow cases were divided into 11 clusters or 20 pulsotypes. SCC or milk yield during the follow-up period was compared for the most common clusters (C11 and C15), and for the most common pulsotypes (C11a and C15b) using univariable mixed-effect linear regression models. Such comparisons were also done between the groups less common clusters and rare clusters, as well as between the groups less common pulsotypes and rare pulsotypes. SCC or milk yield (data not shown) did not differ significantly between the two common clusters, or between the groups less common clusters and rare clusters (Figure 1). However, when the common clusters C11 and C15 were combined into one group, and the less common and rare clusters were combined into another group, significant differences in SCC (p = 0.004), but not in milk yield, were found between these two groups (Figure 2). This difference remained significant in the multivariable model where cows with isolates from the less common/rare cluster group had significantly higher SCC (p = 0.009) compared to cows with isolates from the common clusters. Of the other parameters in the SCC model, a decreased milk yield also increased the SCC (p < 0.001). None of the other tested parameters had any significant effect on the SCC. The geometric mean of SCC during the follow-up period was 123 229 cells/ml for cows with isolates belonging to the common clusters, and 272 351 cells/ml for cows with isolates belonging to the less common/rare clusters.

The proportion of cows with SCC <200 000 cells/ml at all test milkings 14 to 120 days after VTCM was 21% in the group infected with less common/rare clusters (95% CI: 4.6-37.1%). This was significantly lower than in the group infected with the common clusters (51%, 95% CI: 39.4-62.0%).

No significant differences in SCC were found between any of the pulsotypes or pulsotype groups.

### Outcome as measured by treatment failure

Disease recordings were available for 128 cows and culling records for 127 cows of the 133 isolates available for the outcome study. Of these, only 27 cows had one or more registered VTCMadd, and/or were culled during the follow-up period. Because of the small number of observations, comparisons were made only between common clusters combined and less common/rare clusters combined. A larger proportion of cows infected with the less common/rare clusters (31.4%, 95% CI: 16.0-46.8%) had a VTCMadd registered or were culled than cows infected with the common clusters (17.6%, 95% CI: 9.8-25.4%). However, the difference was not statistically significant (p = 0.090).

## Discussion

In the present study of epidemiologically independent *S. aureus* isolates from veterinary treated cases of clinical mastitis in Swedish dairy cows, we found a large genetic variety with 18 clusters divided into 29 pulsotypes. However, two pulsotypes (C11a and C15b) dominated, and together they constituted about two thirds of the studied isolates. Since the isolates were collected in a national survey on the distribution of udder pathogens, and were epidemiologically independent, the distribution of genotypes presented in this study can be considered representative for the country. In a recent publication, based on the same material, but where only a selection of the isolates from certain Swedish regions was included, the same two dominating pulsotypes were found to cause a majority of the mastitis cases [15]. In that study, however, the order of occurrence of the two most common pulsotypes was the opposite of the findings in the present study. The differences in prevalence between the studies could be due to the difference in sample size. It is also possible that it can be explained by the differences in distribution of pulsotypes between Swedish regions that was shown by Capurro *et al.*[15], thus indicating that it can be difficult to draw any conclusions on genotype distributions on local level from a national survey and vice versa.

Also, in several other studies a few predominating strains associated with most intramammary infections in a region have been identified [9,10,12,17]. The reason for a few strains becoming predominant in a region or nation is not known, but it has been speculated that trade of animals can cause a certain genotype to become widespread [15] and that certain genotypes could also have specific traits that increase their chance of spread [17,27].

The results of the present study indicate that bacterial genotype influences the long-term outcome of clinical mastitis caused by *S. aureus.* In cows infected with the most common genotypes, but not in cows infected with less common/rare genotypes, the inflammatory response in the udder, measured by SCC, quickly declined after treatment. In those cases, the SCC was significantly lower following VTCM, and the likelihood of having a SCC <200 000 cells/ml was greater during the follow-up period. The reasons behind the differences between genotype groups are not known. It may be speculated that differences in virulence factors or host adaptation between strains may influence the risk of spread. Another hypothesis may be that the widespread genotypes, instead of being eliminated, became persistent with a low-grade inflammatory response in the udder as discussed in Capurro *et al.*[28]. Such cows might be wrongly assumed to be cured. Follow-up milk sampling for bacterial culture to support the hypothesis on chronic low-grade infections was, however, not possible. In some cases of clinical *S. aureus* mastitis the infected quarter may become non-functional. If such cows remain in the milking herd the cow SCC, based only on milk from remaining healthy quarters, may wrongly indicate that the treatment was successful. Unfortunately, data on presence of non-functional quarters was not available in this study.

We performed statistical analyses on two levels of similarity between isolates, *i.e.* based on clusters (>80% similarity) or pulsotypes (100% similarity). Significant differences in SCC between genotype groups could not be found when using the higher level of similarity, but at cluster level the SCC differed between common clusters and less common/rare clusters. This is in accordance with a study from Finland [19] where differences between *S. aureus* pulsotypes were found at a level of similarity of about 80%. The way of grouping genotypes might explain why no significant differences between genotypes or genotype groups were found when using the higher level of similarity in the present study. When pulsotype prevalence was used to group cows to evaluate outcome, the group “less common pulsotypes” primarily consisted of pulsotypes C3a, C15a and C15c (Table 1). Cows infected with pulsotypes C3a had high SCC in the follow-up period, but pulsotypes C15a and C15c had low SCC (results not shown). When instead clustering the pulsotypes (>80% similarity), pulsotypes C15a and C15c formed the common cluster C15 together with C15b. This resulted in more uniform cluster groups than the heterogeneous pulsotype groups. We would have preferred to compare pulsotypes or clusters individually, but due to the limited study material and the large number of pulsotypes identified, this was not possible.

In the present study, long-term outcome was evaluated using SCC, culling, milk yield and new cases of mastitis up to four months after treatment. Long-term outcome may, however, also be evaluated by studying other parameters or by using a longer follow-up period, but this was not possible in this study.

Treatment of the cows was not standardized or recorded in the original study design. All isolates included in the study were collected from naturally occurring clinical cases of mastitis treated by veterinarians, and treatment was carried out according to the routines of the individual veterinary practices. Because treatment was not standardized and information about individual treatment could not be included in the statistical model, it is possible that different treatments could have an effect on the outcome of individual cases and therefore on the results of this study. However, parenteral treatment with benzylpenicillin is the recommended treatment for cases of clinical mastitis caused by non-β-lactamase producing *S. aureus* in Sweden [29]. At the time when the isolates in this study were collected, 83% of all clinical cases of mastitis in Sweden were treated with parenteral benzylpenicillin [30]. Among the *S. aureus* isolates included in the present study 6.5% produced β-lactamase. Possible treatments for β-lactamase producing *S. aureus* at this time were supportive treatment only, or treatment with spiramycin. As low cure rates for β-lactamase producing *S. aureus* have been reported [7,8], β-lactamase production was included in the statistical model, but was not found significantly associated with any of the outcomes and was therefore not included in the final model.

## Conclusions

In Swedish dairy herds, a few *S. aureus* genotypes were widespread with two pulsotypes identified in 64% of veterinary-treated cases of clinical *S. aureus* mastitis. The long-term outcome of the mastitis cases differed between genotypes. Cows infected with the most common *S. aureus* genotypes had significantly lower SCC in the follow-up period of 120 days after treatment compared to cows infected with less common or rare genotypes.

## Abbreviations

C: Cluster; PFGE: Pulsed-field gel electrophoresis; SCC: Somatic cell count; VTCM: Veterinary-treated clinical mastitis; VTCMadd: Additional episodes of VTCM for the same cow.

## Competing interests

The authors declare that they have no competing interests.

## Authors’ contributions

All authors participated in the design of the study. ÅL performed the genotyping, carried out the statistical analyses and drafted the manuscript. AA participated in genotyping of the bacterial isolates. AN participated in collecting and managing the data and in the statistical analyses. HEU participated in the bacteriological analyses. KPW coordinated the study and helped draft the manuscript. All authors read and approved the final version of the manuscript.
